# Can fecal calprotectin levels be used to monitor infant milk protein allergies?

**DOI:** 10.1186/s13223-021-00636-0

**Published:** 2021-12-13

**Authors:** Liyan Qiu, Junli Wang, Fang Ren, Lixiao Shen, Feng Li

**Affiliations:** Department of Developmental Behavioral Pediatric and Children Healthcare, MOE-Shanghai Key Laboratory of Children’s Environmental Health, Xinhua Hospital, School of Medicine, Shanghai Jiao Tong University, 1665 Kongjiang Rd, Shanghai, 200092 China

**Keywords:** Milk protein allergy, Fecal calprotectin, Growth indexes

## Abstract

**Background:**

Milk protein allergy is one of the most common food allergies in infants. We aimed to test whether fecal calprotectin can be used to monitor food allergies in infants by comparing the fecal calprotectin levels in infants with a milk protein allergy before and after an intervention treatment.

**Methods:**

The study was designed as a prospective case–control trial. Stool samples were collected at follow-up, and the concentration of fecal calprotectin was determined using an enzyme-linked immunosorbent assay. The infant’s weight and length were measured.

**Results:**

The allergic group comprised 90 milk-allergic infants (41 boys, 49 girls), and the nonallergic group comprised 90 nonallergic infants (51 boys, 39 girls). Compared with the fecal calprotectin level in the nonallergic group (median: 141 μg/g), that in the allergic group (median: 410 μg/g) was significantly higher (z = − 9.335, *p* < 0.001). After two dietary interventions and treatments, the fecal calprotectin levels of the infants with a milk protein allergy at the first (median: 253 μg/g) and second follow-up visits (median: 160 μg/g) were significantly lower than those before the intervention (z = − 7.884, *p* < 0.001 and z = − 8.239, *p* < 0.001, respectively). The growth index values (LAZ and WAZ) of the infants with a milk protein allergy at the first and second follow-up visits were significantly higher than those before dietary intervention (*p* < 0.05). Fecal calprotectin was negatively and significantly correlated with the WLZ and WAZ at the second follow-up visit (Spearman’s rho = − 0.234, *p* = 0.01 and Spearman’s rho = − 0.193, *p* = 0.03, respectively).

**Conclusion:**

The level of fecal calprotectin in infants with a milk protein allergy decreased after dietary intervention and seems to be a promising biological indicator for monitoring intestinal allergies.

## Background

Food allergies have a serious impact on the physical and mental health of children, affecting their growth and development and reducing their quality of life and learning. More than 10% of infants under the age of 1 have been shown to have allergic reactions to at least one common allergenic food, and a milk protein allergy is one of the most common food allergies, with an incidence of 2–3% [1]. Milk protein allergies have become a public health problem worldwide, affecting approximately 8% of children [2]. In Europe and the United States, the prevalence of food allergies in children ranges from 5 to 10%, and the prevalence of food allergies in children between 0 and 2 years old in China is 6.2% [3–5]. Food allergies are often considered the first step in the process of allergies [6]. With age, food allergies are more likely to cause serious allergic diseases, such as asthma. Therefore, early diagnosis of and intervention for food allergies in infants and young children will help prevent the further development of allergic diseases, but there are no early predictive indicators for monitoring infant food allergies. The clinical manifestations of children with a milk protein allergy lack specificity; therefore, this type of allergy is easily misdiagnosed or is not diagnosed in a timely manner. The current diagnostic tests are mainly as follows: sIgE test, skin prick test (SPT), patch test and double-blind, placebo-controlled food challenge test. A recent meta-analysis [7] showed that the patch test has a sensitivity of 53% and a specificity of 88%, the SPT has a sensitivity of 88% and a specificity of 68%, and the sIgE test has a sensitivity of 87% and a specificity of 48%. A double-blind, placebo-controlled food challenge test is the gold standard for diagnosing a milk protein allergy. However, it is a complicated test for physicians to perform in the clinic and unsuitable for early diagnosis [8]. Early diagnosis and intervention of food allergies in infants and young children help prevent the further development of allergic diseases, but there is a lack of cost-effective allergy markers. Many researchers are working on biological indicators that can be used to predict and monitor food allergies in infants early, and there is an urgent need for a noninvasive, inexpensive, simple and sensitive method of detecting monitoring the occurrence and development of intestinal inflammatory diseases [9]. A milk protein allergy in infants mainly manifests as skin, digestive and respiratory symptoms. Among them, infants’ gastrointestinal (GI) symptoms are more common and severe. Fecal inflammatory biomarkers, such as calprotectin in infants with an allergy to cow’s milk protein, have been taken into consideration [10–15]. Fecal calprotectin is a simple marker for detecting inflammatory activity in the GI tract and can be used to screen for intestinal diseases [16]. When inflammation occurs in the body, the level of calprotectin can reach 5–40 times the normal level, and the level of fecal calprotectin can reach approximately 6 times the level of calprotectin. Fecal calprotectin is detected by an enzyme-linked immunosorbent assay, which is fast, simple and reproducible and thus fecal calprotectin can be used as a noninvasive, inexpensive, simple and sensitive marker of intestinal inflammation [9, 17]. In our previous study, fecal calprotectin, as an inflammatory factor, was shown to possibly play an important role in food allergy detection [15]. In this study, we aimed to test whether fecal calprotectin can be used as a noninvasive and sensitive biological marker in infants with food allergies by comparing the fecal calprotectin levels of infants with a milk protein allergy before and after intervention treatment and to determine its value in monitoring infants with food allergies in China.

## Methods

### Research design and methodology

The study was designed as a prospective case–control trial with two follow-ups. From September 2019 to August 2020, infants attending the Department of Developmental and Behavioral Pediatric and Child Healthcare of Xinhua Hospital affiliated with Shanghai Jiao Tong University School of Medicine were consecutively invited to participate. We prospectively enrolled infants who were diagnosed with a milk protein allergy and aged 0–9 months as the allergic group. During the study enrollment period, age- and sex-matched controls aged 0 to 9 months, as the nonallergic group, were recruited among healthy infants who underwent regular checkup at the department and did not exhibit allergy or disease symptoms. According to the criteria for the diagnosis of food allergies [11, 18, 19], infants with a milk protein allergy were enrolled in the allergic group, and healthy infants without a milk protein allergy were enrolled in the nonallergic group. Stool collection, physical development assessments, feeding questionnaires and physical examinations were performed during recruitment and follow-up. Ninety infants diagnosed with a milk protein allergy were treated with a dietary intervention as follows [20, 21]. First, milk protein was eliminated from the mother’s diet by promoting a dairy free diet in mothers who exclusively breast-fed their infant. Second, infants for whom the maternal milk protein elimination diet was ineffective received deep hydrolyzed protein milk formula. Third, ordinary milk powder was replaced with extensively hydrolyzed milk formula. Last, an amino acid-based formula (AAF) was given for when the extensively hydrolyzed protein formula was not tolerated. This children attended two follow-ups; the first follow-up was performed approximately one month after the dietary intervention, and the second follow-up was performed approximately two months after the dietary intervention. The parents of two infants refused to participate in the study before enrollment. All the parents of the infants diagnosed with a milk protein allergy were compliant with the dietary intervention after enrollment. In this study, 20 infants received the first type of dietary intervention, 23 infants received the second type, 30 infants received the third type, and 17 infants received the fourth type. Stool collection, physical development assessments, feeding questionnaires and physical examinations were performed at each follow-up.

### Study population: inclusion criteria and exclusion criteria

The infants recruited in the allergic group and the nonallergic group were matched for age, sex, and socioeconomic status. All the recruited children met the following inclusion criteria: birth weight appropriate for gestational age (2500 ~ 4000 g); no illnesses in the month prior to enrollment; and no known underlying chronic inflammatory disease. The diagnosis of a food allergy was conducted according to recommendations for the diagnosis and treatment of infantile food allergies [11, 19, 22]. The diagnosis of a food allergy was indicated by medical history and physical examination results and was confirmed by medical history, physical examination, clinical manifestations, an SPT, a food elimination test (elimination diet) and food challenge tests (food challenge). When clinical symptoms (diarrhea, abdominal pain, hematochezia, urticaria etc.) indicative of a milk protein allergy were observed, the infants were prevented from consuming milk protein in the diet for two weeks, and an oral food challenge test was subsequently conducted. The oral challenge was considered to be positive when there were skin (urticaria, angioedema or erythematous rash), digestive (vomiting or diarrhea), respiratory (rhino-conjunctivitis or bronchospasms) or generalized (anaphylactic shock) manifestations after the intake of the milk formula [23, 24], and the diagnosis of milk protein allergy was confirmed [25]. After a positive oral food challenge test, stool samples of infants with a diagnosis of milk protein allergy were collected for testing. The exclusion criteria were as follows: any intake of steroidal or nonsteroidal anti-inflammatory drugs, gastric acidity inhibitors, antibiotics or any other drug during the 2 weeks prior to recruitment; nasal bleeding during the week before the study; or a history of signs or symptoms of infection or gastrointestinal disease (diarrhea, vomiting, hematochezia and fever).

### Anthropometric measurements and calculations

Each infant’s weight and supine length were measured using standard techniques. Anthropometric measurements of the infants were performed in duplicate by a trained member of the research team as described in our previous study [9, 16, 26, 27]. The length-for-age Z-score (LAZ), weight-for-age Z-score (WAZ), and weight-for-length Z-score (WLZ) were calculated using Anthro software (version 3.1) based on the World Health Organization Child Growth Standards.

### Questionnaire

At enrollment, the parents of the children were asked to complete a brief health questionnaire regarding several clinical and sociodemographic factors. Clinical features, including gestational age, birth weight, sex, neonatal diseases, symptoms, physical examination findings, feeding status, weight and length, were recorded prior to the collection of each stool sample.

### Fecal calprotectin measurement

The fecal calprotectin concentrations of the infants with an allergy to cow’s milk protein at the first follow-up visit and the second follow-up visit were determined. A parent of each child was provided with a plastic container and was instructed on how to collect a stool sample. The parents removed a fecal sample from their child’s diaper, and the sample was brought or sent in a screw-capped container to the hospital. All fecal samples were frozen and stored at − 80 °C immediately following receipt until analysis. The calprotectin concentration in each sample was determined using a commercially available enzyme-linked immunosorbent assay (ELISA) that quantitatively measures calprotectin levels (Bühlmann Laboratories AG, Schönenbuch, Switzerland) as previously described [9, 16, 26]. Included in each sample run were blanks, standards and controls. Prior to analysis, frozen stool samples were thawed at room temperature. If the sample yielded a reading greater than the maximum calibrated level (600 μg/g), the remaining extract of the sample was further diluted 1:6 with incubation buffer, and the assay was repeated. Calprotectin levels are expressed as μg/g of feces. Informed consent was obtained from parents at enrollment. The study protocol was approved by the ethics committee of Xinhua Hospital affiliated with Shanghai Jiao Tong University School of Medicine.

## Results

### General characteristics

The study recruited 90 milk-allergic infants (41 boys, 49 girls) as the allergic group and 90 nonallergic infants (51 boys, 39 girls) as the nonallergic group. The average length of the 90 infants in the nonallergic group was 64.17 cm, and the average weight was 7215 g. Among the 90 infants in the allergic group, the median gestational age was 39 weeks (range 37–42 weeks), and the average weight at birth was 3256 g (range 2500–4000 g). The average length of the 90 infants in the allergic group before dietary intervention was 63.79 cm, and the average weight was 6790 g. There were significant differences in the weight of the infants between the allergic group and the nonallergic group (t = − 2.047, *p* = 0.04). There was no significant difference between the allergic group and the non-allergic group in the maternal pregnancy age, birth weight or age. Among the 90 infants in the allergic group, the main clinical features were diarrhea (n = 58), abdominal pain (n = 45), hematochezia (n = 53), vomiting (n = 22), urticaria (n = 11), and constipation (n = 3) (Table 1).

**Table 1:** Characteristics of the infants

Characteristic	Milk allergy (N = 90)	Non-milk allergy (N = 90)	F/χ^2^	*p*
Boys/girls	41/49	51/39	2.223	0.179
Gestational age (weeks, mean ± SD)	39.1 ± 1.2	38.8 ± 1.0	1.462	0.228
Birth weight (g) (mean ± SD)	3256 ± 383	3253 ± 384	0.467	0.495
Weight at sample collection (g)	6792 ± 1432	7215 ± 1338	− 2.047	0.042
Length at sample collection (cm)	63.7 ± 4.7	64.2 ± 4.4	− 0.642	0.522
Age				
0–3 months	20 (22.2%)	20 (22.2%)	103.310	0.240
3–6 months	47 (52.2%)	47 (52.2%)		
6–9 months	23 (25.6%)	23 (25.6%)		
Clinical features				
Diarrhea	58 (64.4%)	–	–	–
Hematochezia	53 (58.9%)	–	–	–
Abdominal pain	45 (50.0%)	–	–	–
Vomiting	22 (24.4%)	–	–	–
Urticaria	11 (12.2%)	–	–	–
Constipation	3 (3.3%)	–	–	–

*p* < 0.05 was considered significant

### Comparison of fecal calprotectin before and after dietary intervention and treatment in infants with a milk protein allergy

**Fig. 1:**
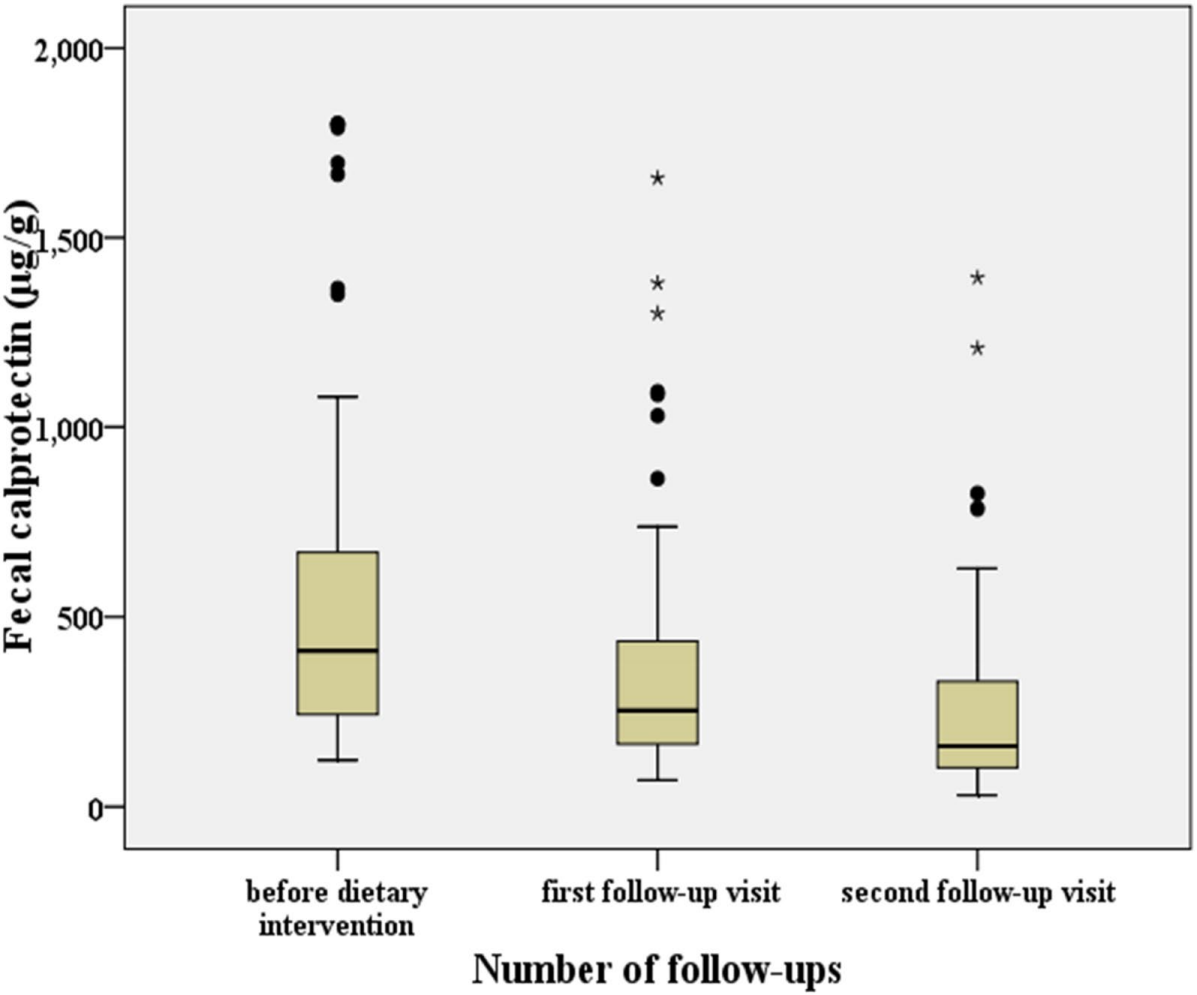
Comparison of fecal calprotectin levels of infants with a milk protein allergy before dietary intervention and at the first and second follow-ups

The median fecal calprotectin level for the 90 infants in the nonallergic group was 141 μg/g feces (interquartile range: 41–373 μg/g). The median fecal calprotectin level of the 90 infants in the allergic group was 410 μg/g (interquartile range: 168–1739 μg/g) before dietary intervention. Compared with the nonallergic group, the fecal calprotectin level in the allergic group was significantly higher (z = − 9.335, *p* < 0.001). After dietary intervention and treatment for approximately one month, the median value of fecal calprotectin at the first follow-up visit was 253 μg/g (interquartile range: 105–1089 μg/g). After approximately two months of dietary intervention, the median value of fecal calprotectin at the second follow-up visit was 160 μg/g (interquartile range: 34–699 μg/g). Compared with those before intervention, the fecal calprotectin levels of the infants with a milk protein allergy at the first and second follow-up visits decreased significantly (z = − 7.884, *p* < 0.001 and z = − 8.239, *p* < 0.001, respectively). Compared with those at the first follow-up visit, the fecal calprotectin levels of the infants with a milk protein allergy also decreased significantly at the second follow-up visit (z = − 8.173, *p* < 0.001; Table 2 and Fig. 1).

**Table 2:** Comparison of fecal calprotectin levels in infants with a milk protein allergy before and after intervention (n = 90)

	Nonallergic group	Allergic group			*Wilcoxon	*p	^#^Wilcoxon	^#^*p*	^※^Wilcoxon	^※^*p*
		Before intervention	First visit	Second visit						
FC (median, 5th–95th, μg/g)	141 (41–373)	410 (168–1739)	253 (105–1089)	160 (34–699)	− 7.884	< 0.001	− 8.239	< 0.001	− 8.173	< 0.001
Eosinophils (median, 5th–95th, 106)	50 (50–648)	655 (231–1770)	495 (235–1129)	330 (161–828)	− 6.679	< 0.001	− 7.959	< 0.001	− 7.962	< 0.001

FC: fecal calprotectin; 5th–95th: 5–95 percentile

*p* < 0.05 was considered significant

*Comparison between the first follow-up visit and before the intervention, ^#^Comparison between the second follow-up visit and before the intervention, ^※^Comparison between the second and the first visit

### Comparison of the eosinophil count before and after dietary intervention and treatment in infants with a milk protein allergy

**Fig. 2:**
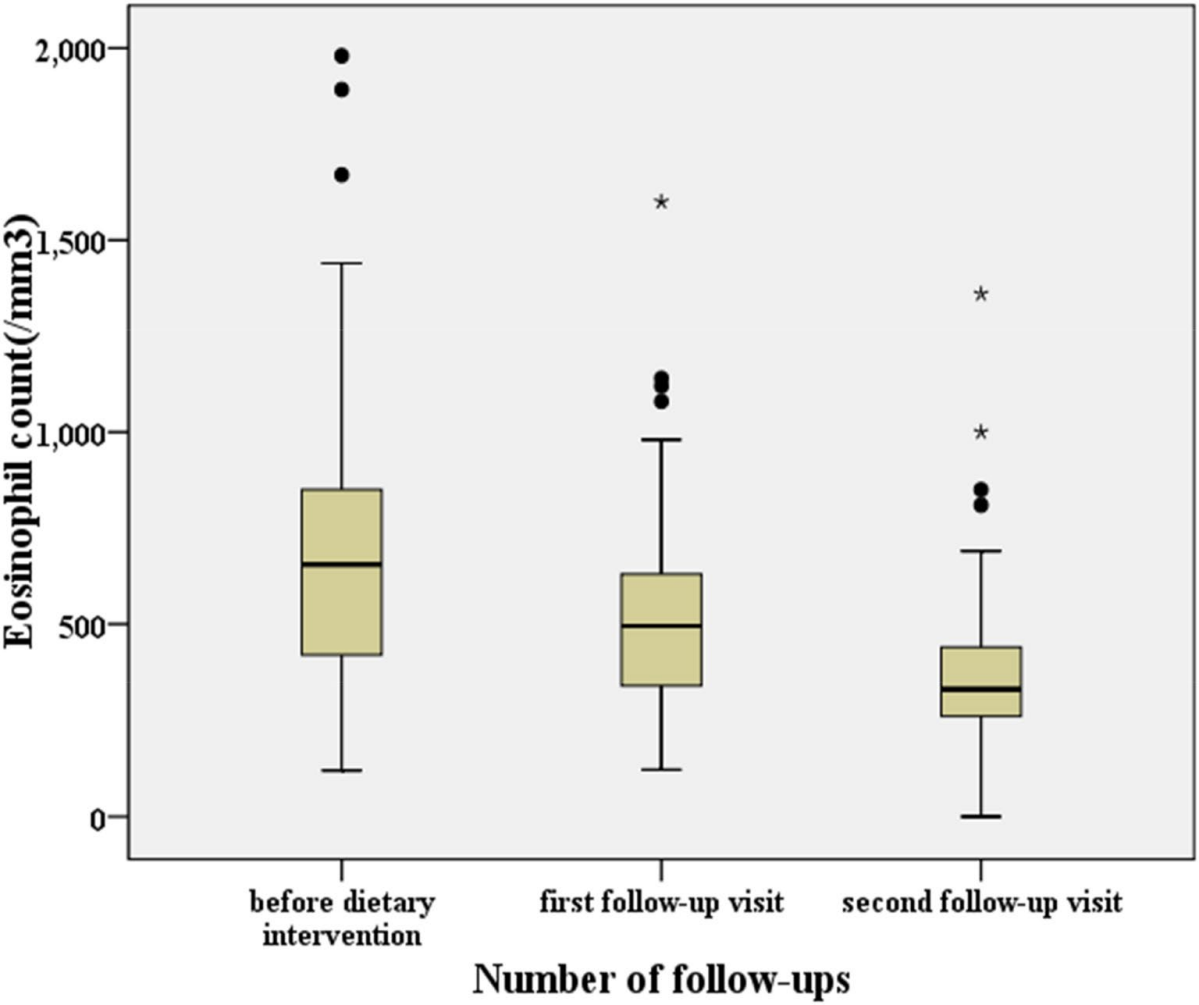
Comparison of the eosinophil count of infants with a milk protein allergy before dietary intervention and at the first and second follow-ups

The median eosinophil count for the 90 infants in the nonallergic group and the 90 infants in the allergic group was 50/mm^3^ (interquartile range: 50–648/mm^3^) and 655/mm^3^ (interquartile range: 231–1770/mm^3^), respectively, before dietary intervention. Compared with the nonallergic group, the eosinophil count of the allergic group was significantly higher (z = − 9.505, *p* < 0.001). After dietary intervention and treatment for approximately one month, the median eosinophil count at the first follow-up visit was 495/mm^3^ (interquartile range: 235–1129/mm^3^). After two months of dietary intervention, the median eosinophil count at the second follow-up visit was 330/mm^3^ (interquartile range: 161–828/mm^3^). Compared with those before dietary intervention, the eosinophil counts of the infants with a milk protein allergy at the first and second follow-up visits decreased significantly (z = − 6.679, *p* < 0.001 and z = − 7.959, *p* < 0.001, respectively). Compared with those at the first follow-up visit, the eosinophil counts of the infants with a milk protein allergy were also significantly decreased at the second follow-up visit (z = − 7.962, *p* < 0.001; Table 2 and Fig. 2). A simple correlation analysis of the number of eosinophils and fecal calprotectin showed that the number of eosinophils and the level of fecal calprotectin were positively correlated and significantly correlated before the intervention treatment and at the first and second follow-up (Spearman’s rho = 0.447, *p* < 0.001; Spearman’s rho = 0.487, *p* < 0.001; Spearman’s rho = 0.474, *p* < 0.001, respectively).

### Comparison of growth indexes of infants with a milk protein allergy before and after dietary intervention and treatment

**Fig. 3:**
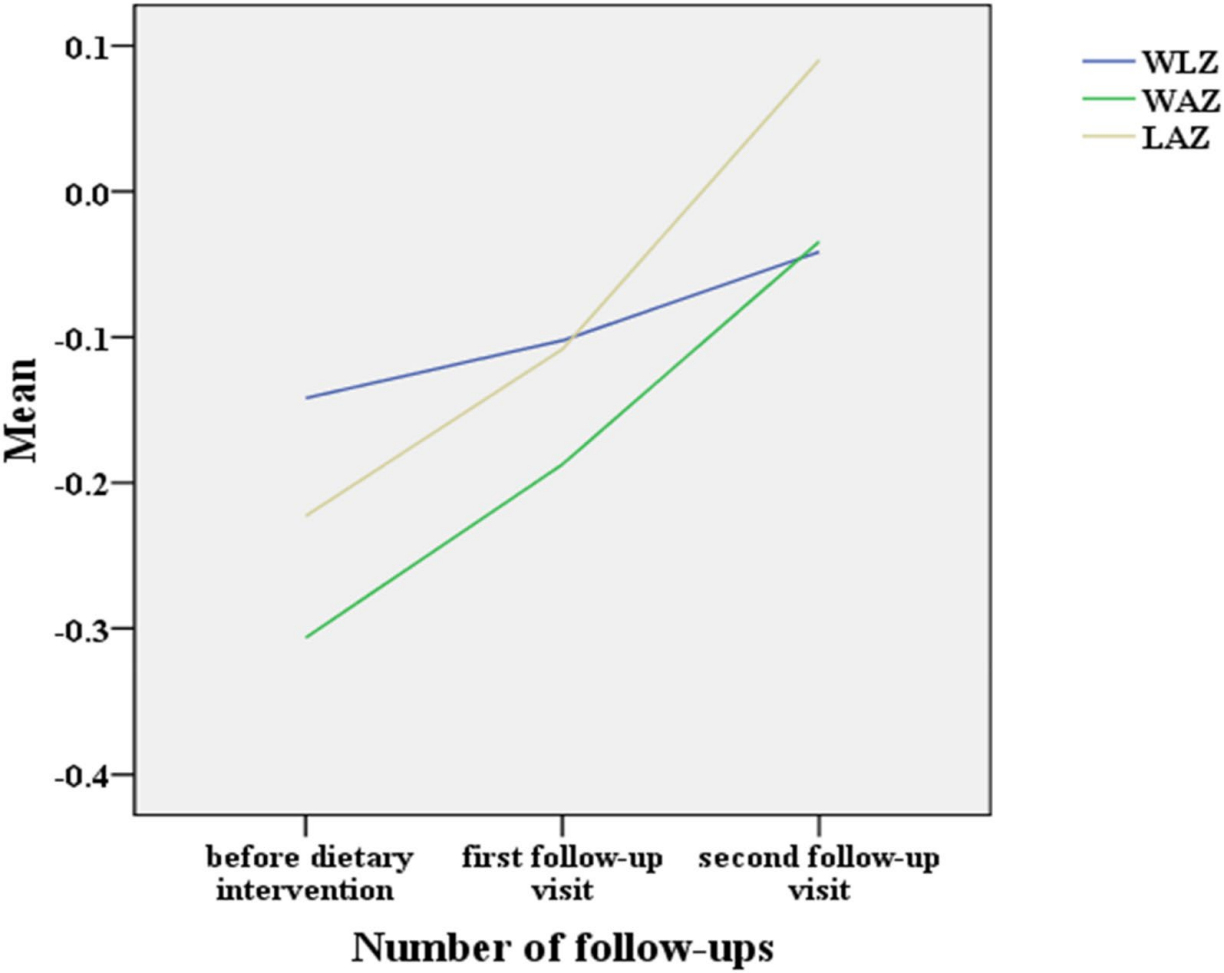
Changes in LAZ, WAZ, and WLZ in infants with a milk protein allergy before dietary intervention and at the first and second follow-ups

According to the data analysis results, the LAZ and WAZ values of the infants with a milk protein allergy at the first follow-up visit were significantly higher than those before dietary intervention (t = − 3.318, *p* = 0.001 and t = − 3.619, *p* < 0.001, respectively). The change in the WLZ was not significant (t = − 1.083, *p* = 0.279). After two months of intervention and treatment, the LAZ and WAZ values of the infants with a milk protein allergy at the second follow-up visit increased significantly compared with those before dietary intervention (t = − 3.298, *p* = 0.001 and t = − 4.472, *p* < 0.001, respectively). The LAZ and WAZ values of the infants with a milk protein allergy at the second follow-up visit were significantly higher than those at the first follow-up visit (t = − 3.016, *p* = 0.003 and t = − 3.932, *p* < 0.001, respectively). The values of LAZ, WAZ and WLZ from before the dietary intervention to the follow-up to the intervention increased as shown in Table 3 and Fig. 3. A simple correlation analysis of the WLZ and WAZ with the fecal calprotectin level showed that the level of fecal calprotectin was negatively and significantly correlated with the WLZ and WAZ before dietary intervention (Spearman’s rho = − 0.204, *p* = 0.006 and Spearman’s rho = − 0.228, *p* = 0.002, respectively) and that the fecal calprotectin level was negatively and significantly correlated with the WLZ and WAZ at the second follow-up visit (Spearman’s rho = − 0.234, *p* = 0.01 and Spearman’s rho = − 0.193, *p* = 0.03, respectively).

**Table 3:** Comparison of growth and development of infants with a milk protein allergy before and after intervention (n = 90)

	Nonallergic group	Allergic group			*t	*p	^#^*t*	^#^*p*	^※^*t*	^※^*p*
		Before intervention	First visit	Second visit						
WLZ (Mean ± SD)	0.365 ± 1.013	− 0.142 ± 1.091	− 0.102 ± 0.932	− 0.041 ± 0.831	− 0.881	0.380	− 1.897	0.061	− 1.899	0.061
WAZ (Mean ± SD)	0.315 ± 0.786	− 0.306 ± 1.171	− 0.187 ± 0.957	− 0.034 ± 0.852	− 1.837	0.070	− 3.323	0.001	− 3.759	< 0.001
LAZ (Mean ± SD)	0.135 ± 0.891	− 0.223 ± 1.216	0.108 ± 0.970	0.090 ± 0.921	− 1.164	0.248	− 2.598	0.011	− 3.023	0.003

*p* < 0.05 was considered significant

*LAZ* length-for-age Z-score, *WAZ* weight-for-age Z-score, *WLZ* weight-for-length Z-score

*Comparison between the first follow-up visit and before the intervention, ^#^Comparison between the second follow-up visit and before the intervention, ^※^Comparison between the second and the first visit

## Discussion

Infant food allergies are a serious and often life-threatening health problem, affecting approximately 4% of children and their families worldwide [22]. These allergies may cause anaphylactic shock or even death, but there is currently no drug therapy for a milk protein allergy. The only way to avoid this type of allergy is to eliminate the harmful milk protein from the diet [28, 29]. Management strategies for a milk protein allergy include removing dairy products from exclusively breastfed babies and eliminating milk protein from the mother’s diet. Breast milk is a source of “standard nutrition” for infants with milk protein allergies and should be promoted as much as possible [30]. Data on the prevalence of reproducible clinical responses to milk protein in breastfed children are very limited, but the prevalence has been reported to be approximately 0.5% [31]. Although b-lactoglobulin originating from cow’s milk can be detected in the breast milk of 95% of lactating women, the amount is insignificant to many infants with a mild to moderate milk protein allergy [32]. For babies who are allergic to milk protein from breastfeeding, mothers should be encouraged to continue breastfeeding while avoiding milk protein in their diet [29]. However, if breastfeeding or maintenance of breastfeeding is not possible in patients with a milk protein allergy, a hydrolyzed milk formula or amino acid formula should be provided.

If the symptoms of infants who are breastfed or fed only formula milk cannot be relieved, it is recommended that extensively hydrolyzed formula milk is used. Hypoallergenic products are either extensively hydrolyzed milk powder (ehMF) composed of small peptides < 1.5 kDa or amino acid formulas composed of essential and nonessential amino acids. The latter is recommended for intolerant infants affected by ehMF [30, 33]. Amino acid-based formulas (AAFs) are usually used to address complex milk protein allergies, including various food allergies or formulas that do not tolerate large amounts of hydrolysis. In our study, infants with a milk protein allergy were treated with a dietary intervention (involving amino acid formulas or deeply hydrolyzed formulas or mothers avoiding the consumption of milk), and the allergic symptoms gradually decreased.

Calprotectin reflects the migration of neutrophils to the intestinal lumen, so it can be used as a sensitive marker of intestinal inflammation [17]. Under normal circumstances, its concentration in feces is six times the concentration in plasma [34], emphasizing the potential of fecal calprotectin as an accurate biomarker of intestinal inflammation. The evidence supporting the use of fecal calprotectin as a marker of intestinal inflammation is sufficient and continues to accumulate [35]. In addition, fecal calprotectin remains stable in the feces for more than a week, so it is a useful marker of intestinal inflammation [36] and can be measured within a few hours using a simple ELISA test; therefore, the test results are quickly available for effective clinical decision making. Calprotectin has attracted increasing attention in studies of food allergies [10, 14, 37, 38]. Fecal calprotectin may play an important role in food allergies, and it is speculated that in addition to being an inflammatory factor, calprotectin has a role in the process of food allergies, possibly as a trigger that amplifies the cascade reaction of allergic-related and inflammatory factors in allergic responses [15]. In response to food allergens, eosinophils and neutrophils are activated, while neutrophils and epithelial cells in the intestinal mucosa activate calprotectin, resulting in increased levels of calprotectin [15]. Most studies have indicated that the activation of Th2 cells is an important step in the immune mechanism of food allergies [15, 39, 40]. In the process of allergies, there are inflammatory features of the Th2 cytokine environment (such as increased eosinophils and mast cells) and local eosinophilia during allergies. The activation of granulocytes and neutrophils is recruited and activated. It can be speculated that this may lead to an increase in the expression and secretion of calprotectin, which indicates that there may be a process similar to inflammation in an allergic state [41]. Our previous study indicated that calprotectin may activate DCs and related signal transduction through the action of TLR4 to promote the differentiation of initial CD4 + T cells into Th2-type cells and then cause allergy-related immune responses, leading to the occurrence of allergies. The increase in S100A8/A9 amplifies the cascade of allergic and inflammatory factors in food allergies [15]. Baldassarre et al. [42] compared the fecal calprotectin levels of 30 milk protein allergic infants with rectal bleeding and healthy infants of the same age. The fecal calprotectin level of infants with a milk protein allergy was significantly higher than that of the control group (325.89 vs. 131.97 µg/g). Four weeks after removing milk from the diet, the fecal calprotectin level dropped by 50%; however, it was still higher than that of the control group (157.5 vs. 93.72, *p* = 0.03). Beşer et al. [13] found that infants who were allergic to milk protein had significantly lower fecal calprotectin levels after eliminating milk protein from the diet than before eliminating milk protein from the diet. In addition, the level of fecal calprotectin before the milk protein elimination diet was significantly higher than that of healthy infants (*p* = 0.011). In a preliminary study of 6 patients with a milk allergy [13], fecal calprotectin levels were measured before dietary intervention and at 3 and 6 weeks after the initiation of protein hydrolysate formula feeding. The fecal calprotectin level before dietary intervention was 135–1537 mg/L (average: 557 mg/L) and decreased to 42–219 mg/L (average: 163 mg/L) after six weeks. The fecal calprotectin level of infants with a milk protein allergy was compared with that before treatment; after treatment, the fecal calprotectin level decreased, and the clinical symptoms were alleviated [13], which is consistent with our results. In this study, the median fecal calprotectin values of 90 infants with a milk protein allergy before intervention and at the first and second follow-up visits were 410 μg/g, 253 μg/g and 160 μg/g, respectively. With the extension of dietary intervention and treatment time, allergic symptoms improved, and the level of fecal calprotectin in infants with a milk protein allergy gradually decreased. This suggested that fecal calprotectin may be useful for determining relapses and follow-ups after diagnosis of a milk protein allergy, particularly an allergy with GI involvement. Tracking fecal calprotectin levels might reveal increases or reductions in disease activity, and it may be useful as an inexpensive, simple, and noninvasive test to demonstrate and assess disease activity in infants with a milk protein allergy. The level of fecal calprotectin may be used to monitor the improvement in intestinal allergies in infants with a milk allergy, which may be used as a possible biological indicator for follow-up and monitoring of intestinal allergies.

In this study, the number of eosinophils in the infants with a milk protein allergy was much greater than that in the infants with a non-milk protein allergy, and with dietary intervention and treatment, the number of eosinophils in the allergic infants gradually decreased and tended to be normal. We found that the eosinophil count (495/mm^3^) of the infants with a milk protein allergy was significantly lower at the first follow-up visit than before dietary intervention (655/mm^3^) and that the number of eosinophils at the second follow-up visit (330/mm^3^) was significantly lower than that at the first follow-up visit (495/mm^3^). In a similar study by Dogan et al. [43] found significantly higher eosinophil cationic protein levels (51.45 ng/mL) and blood eosinophil counts (475/mm^3^) in infants with a milk protein allergy than in controls (17.55 ng/mL, 300/mm^3^). Recently, Li et al. [44] found that in 6-month-old infants with a milk protein allergy, eosinophil counts were higher than those of the nonallergic group (0.89 ± 0.45/mm^3^ and 0.26 ± 0.12/mm^3^, respectively, *p* < 0.01). All of the above studies have found that infants with a milk protein allergy have an increased number of eosinophils in their blood. The eosinophils formed in the bone marrow have large cytoplasmic granules, which contain eosinophil cationic protein, eosinophil protein X, and eosinophil-derived neurotoxin [43]. The eosinophil cationic protein encoded by the RNASE3 gene is a cytotoxic protein that enters the surrounding tissues when activated eosinophils degranulate and manifests as an increase in the level of eosinophils in the surrounding tissues [45]. Therefore, the response to Th2-induced allergic diseases (such as milk protein allergy, asthma and inflammatory diseases) may increase the number of circulating eosinophils and eosinophil cationic protein levels. Eosinophilic cationic protein is one of the four main basic proteins in specific granules in the cytoplasm of eosinophils. It can reflect the activity of eosinophils and elevated levels in body fluids such as saliva, serum and feces in the course of inflammatory processes and allergic diseases [43]. When inflammation and allergic reactions occur in the body, the level of fecal calprotectin and the number of eosinophils is increased [10, 43, 46], so the level of fecal calprotectin and the number of eosinophils is positively correlated.

In this study, we also recorded the changes in the baby’s weight and length before and after the intervention. From the results of our data analysis, after dietary intervention, the growth indicators of allergic children increased significantly during the first and second follow-ups of the intervention. A study showed that [47] infants allergic to milk protein who received amino acid-based infant formula gained weight and exhibited a decreased allergic performance. Children have poor growth related to low-grade inflammation affecting GI barrier function, leading to suboptimal nutrient absorption [30]. A diet that does not contain milk protein, especially a large amount of hydrolyzed formula, can reduce GI symptoms due to changes in immune mechanisms and exercise capacity (for example, reducing gastric emptying time) [48]. There is evidence that hydrolyzed infant formula may have a long-term preventive effect on the development of allergic symptoms [49]. Our research results are similar to the above published studies, and we found that through a dietary intervention, the height and weight of the child gradually increased, which was better than the levels before the intervention.

This study shows that the fecal calprotectin level of infants with a milk protein allergy is significantly higher than that of healthy infants without an allergy. The level of fecal calprotectin was negatively correlated with the growth and development of the infants with a milk allergy. These infants were treated with a dietary intervention, and the symptoms of intestinal allergies were improved. The level of fecal calprotectin also decreased with the remission of allergic symptoms. The lower the level of fecal calprotectin was, the better the growth and development of the infants with a milk allergy. Fecal calprotectin may be used as a possible marker for monitoring intestinal hypersensitivity in infants. The level of fecal calprotectin may be used to monitor the improvement in intestinal allergy in infants with a milk allergy and may be used as a biological indicator for follow-up and monitoring of intestinal allergy. However, in a recent review by Xiong et al. [10] including thirteen studies with IgE-mediated and non-IgE-mediated milk protein allergies, the authors concluded that the available evidence was not sufficient to confirm the utilization of fecal calprotectin, neither for diagnosis nor for the monitoring of a milk protein allergy. This may be due to minor infections or even non-GI infections that may affect the level of fecal calprotectin. This fact could result in uncertain clinical interpretations if fecal calprotectin is used as a biomarker for milk protein allergy diagnosis in infants, as mild infections are frequent at this age [50]. More studies are needed in the future to determine the value of fecal calprotectin levels in allergic diseases.

### Limitations

Our study has several limitations. First, stool samples were collected from the children’s diapers. Olafsdottir et al. [51] reported that this method of collection increases the fecal calprotectin concentration by up to 30% because water is absorbed into the diaper. This could yield measured fecal calprotectin levels that are higher than those actually present; therefore, direct stool collection during excretion may be more practical [52]. Second, we did not draw blood to detect IgE in the milk protein allergy group, and we could not determine whether the children had IgE-mediated or non-IgE-mediated allergic reactions. Third, we did not follow up with healthy children in this study. For ethical reasons, it was not possible to carry out any test in the control group to rule out the possibility of asymptomatic milk protein allergy. The fecal calprotectin value was not detected before the oral food challenge test. We did not determine the calprotectin levels in patients which confirmed CMPA who developed natural tolerance or in children with an asymptomatic sensitization to milk. This maybe our future work. Finally, this study had a small sample size and short follow-up time, and a large-sample study with a longer follow-up time is needed to investigate the role of calprotectin in the intestinal tract of children with food allergies.

## Conclusion

The level of fecal calprotectin in infants with a milk protein allergy decreased after a dietary intervention. The level of fecal calprotectin may be used to monitor the improvement of intestinal allergies in infants with a milk allergy and may be used as a possible biological indicator for follow-up and monitoring of intestinal allergies.

## Data Availability

The datasets used and analyzed during the current study are available from the corresponding author on reasonable request.

## Abbreviations

LAZ: Length-for-age Z-score
WAZ: Weight-for-age Z-score
WLZ: Weight-for-length Z-score
